# Supplementary material for: Can rhythm-induced attention improve the perceptual representation?
Source: PLoS One. 2020 Apr 16;15(4):e0231200. doi: 10.1371/journal.pone.0231200 (PMC7162507; doi:10.1371/journal.pone.0231200)
Supplement: Attachment — Submitted filename: Response to Reviewers.pdf [file pone.0231200.s002.pdf]

<u>**Response to reviewers**</u>

We would like to thank the reviewers once again for the time and effort they invested in our manuscript. We were happy to learn that only minor comments were left and we addressed all of them. Below is a detailed response to each comment made by each reviewer.

Reviewer #1:

I'm happy with the revisions to the paper. I have a few minor typographical/formatting comments below.

line 143: "due to a too noisy data" – remove "a"

line 144: "analyses are conducted we had" – add a comma after "conducted"

line 176: "50m" – presumably this should be "50ms"

line 186: "trials with which" – replace "with" with "in"

line 216: "with which rhythm type" – replace "with" with "in"

page 11: be consistent with significant digits used to report p values

line 270: follow APA format for reporting F-scores below 1

line 455: "De la Rosa et al. [53] study" – add "The" before "De la Rosa"

Reference section: a number of minor APA formatting issues here, presumably these will be addressed at the copy-editing stage

Response: We fixed all of the above comments as suggested by the reviewer, apart for the comment about the format of the references. To the best of our understanding this format should be Vancouver not APA. We therefore left the references as they are.

Reviewer #2: (No Response)

Reviewer #3: This is the first revision of a previously submitted manuscript. I have only a few minor remarks.

p. 3, line 57 ff: "Based on such rhythm effects, the dynamic attending theory (DAT) suggests that entrainment to a rhythm reflects external rhythms driving internal oscillators leading to a narrower attentional focus with regular (isochronous) rhythms than with irregular (asynchronous) rhythms." This sentence is somewhat confusing and would benefit from rewriting.

Response: We rephrased this sentence and split it into 2 sentences.

p. 6, line 117 ff: "For example, Miller, Carlson, and McAuley [26] have found that an auditory rhythm reduced saccade latency to a visual target and increased discrimination accuracy when target onset was in-phase with the rhythm." Maybe rephrase to "in-phase with the preceding auditory

rhythm”. In addition, it might be good to split the sentences into two sentences and mention more specifically that the visual target could occur either in-phase or out-phase.

Response: Fixed

p. 18, line 432: “Still, such attentional effects may be less robust under cross-modal setting.” It is not entirely clear what the authors would like to say with this sentence.

Response: We added a sentence that clarify what we meant.
